# The association between sedentary behavioral characteristics and poor vision among Chinese children and adolescents

**DOI:** 10.3389/fpubh.2022.1043977

**Published:** 2022-12-05

**Authors:** Lin Li, Jinjin Liao, Hui Fu, Boyi Zong

**Affiliations:** ^1^Key Laboratory of Adolescent Health Assessment and Exercise Intervention of Ministry of Education, East China Normal University, Shanghai, China; ^2^College of Physical Education and Health, East China Normal University, Shanghai, China

**Keywords:** sedentary behavior, sedentary time, sedentary type, children and adolescents, poor vision

## Abstract

**Introduction:**

To understand the features of sedentary behavior of Chinese children and adolescents and its relationship with poor visual acuity, a self-administered “Questionnaire on Sedentary Behavior of Children and Adolescents” was used to survey 4,203 students in grades 4–12 in six administrative regions of China.

**Results:**

(1) The average time spent in sedentary behaviors (SB) of Chinese children and adolescents was about 8.1 h per day, of which the academic sedentary time was the longest, accounting for 79.2% of total sedentary time. The total time spent on SB and the time spent on studying SB were more in the upper grades and less in screen SB and cultural leisure SB, respectively. There were significant sex differences in total SB time (*p* < 0.05) and weekend sedentary behaviors time (SB-WD) (*p* < 0.01) among Chinese children and adolescents, with girls being more likely to be higher than boys. There were also significant differences in sedentary time across different regions (*p* < 0.05), and the longest total sedentary time in East China. (2) Reduction parents' sedentary time and limitation of sedentary behaviors and the use of electronics among children and adolescents can effectively reduce sedentary time among Chinese children and adolescents. (3) Sedentary time was significantly higher in children and adolescents with poor vision than in those with normal vision (*p* < 0.01), and study SB and screen SB were important independent factors affecting vision. (4) Timing of breaks in SB can play a positive role in promoting vision health.

**Conclusion:**

There were significant grade, sex, and regional differences in the SB of Chinese children and adolescents, and sedentary time was strongly related to the prevalence of poor vision detection rate.

## Introduction

As societies become more modernized, people's lifestyles have gradually changed. Sedentary behaviors refer to behaviors that consume energy in the range of 1.0 to 1.5 metabolic equivalents when an individual is sitting or lying in a awake state (1). In recent years, the sedentary behavior of children and adolescents worldwide has become a common phenomenon, although there are slight differences in each country (2–6).

Studies have confirmed that sedentary behavior in children and adolescents is associated with physical activity (7) and other health-related outcomes, such as obesity (8) and cardiovascular disease (9). Notably, sedentary behavior may also be strongly associated with poor visual acuity (10). The longer the sedentary time, the higher the risk of poor vision (11). Studies have found that academic stress and excessive use of electronic devices contribute to vision loss in children and adolescents (12–14). In China, the average school time for children and adolescents is around 9 h and excessive school time leads to increased eye stress. Meanwhile, Chinese children have hand-held screen devices at an early age, and premature exposure to electronic screen devices may result in vision loss. To improve visual acuity of children and adolescents, most schools require students to do eye exercises more than once a day and organize a vision examination per school year. In addition, some parents also take their children to the eye hospital for vision examination and eye care. Recent studies have shown that sedentary school-age children's behaviors, including reading and writing homework, as well as excessive Internet use and television viewing, have been associated with vision loss (13, 15). However, contrary results were also reported. Mountjoy et al. (16) reported that sedentary behaviors like reading are not associated with visual acuity in children and adolescents. Wu et al. have shown that sedentary behaviors such as watching television are also not associated with visual acuity in children (17). Thus, there are few studies and inconsistent findings on the relationship between sedentary behaviors and poor visual acuity in children and adolescents, which makes the relationship difficult to clarify and needs to be further explored.

This study selected students from grades four to 12 in six administrative regions of China, including North China, East China, South Central China, Northeast China, Southwest China and Northwest China (except Hong Kong, Macao and Taiwan), to understand the current situation of sedentary behavior of Chinese children and adolescents and reveal the characteristics of sedentary behavior of Chinese children and adolescents by investigating indicators such as the total time of sedentary behavior, types of sedentary behavior and interval time of sedentary behavior. On this basis, we explore the relationship between sedentary behavior and poor vision of children and adolescents, identify the main factors affecting poor vision caused by sedentary behavior, and hope to open up new ideas for improving the sedentary lifestyle of children and adolescents and promoting eye health of children and adolescents.

## Methods

### Study design and participants

The participants were selected according to the six administrative regions (Northeast China, North China, East China, Northwest China, Southwest China and South Central China) of the country, covering 20 provinces (cities, districts) including Shanghai, Tianjin, Chongqing, Anhui, Fujian, Guangdong, Hebei, Henan, Heilongjiang, Jilin, Jiangsu, Liaoning, Shandong, Sichuan, Zhejiang, Yunnan, Shanxi, Inner Mongolia Autonomous Region, Xinjiang Uygur Autonomous Region, and Tibet Autonomous Region, according to each administrative division plans to distribute 900 students. Considering that the cognitive abilities of children and adolescents in grades one to three are still immature, they do not understand the content of the questionnaire, and have no ability to fill in the questionnaire independently, students in grades four to 12 with an age range of 11 to 17, were selected for the questionnaire. Five thousand four hundred online questionnaires were distributed, and 4,735 questionnaires were returned. The following participants were excluded: (1) the filling attitude was incorrect, which means participants filled in the questionnaire with incomplete content, such as missing questions, wrong lines, no personal information, the answer choices did not meet the filling requirement, such as ticking ≥2 answers for a single choice question, and it was obvious that the questionnaire was filled in randomly; (2) the questionnaire was repeatedly filled. Finally, 4,203 valid questionnaires were obtained, with a recovery rate of 77.8%, including 2,041 boys and 2,162 girls (Table 1). This study was reviewed and approved by the human subject protection committee of East China Normal University (approval No.: HR234-2019). Consents were obtained from all participants and their parents/guardians, and informed consent was signed.

**Table 1:** Region, sex and grade distribution of children and adolescents.

**Region**	**Northeast China**		**North China**		**East China**		**Northwest China**		**Southwest China**		**South Central China**		**Total**
**Grade**	**Boys**	**Girls**	**Boys**	**Girls**	**Boys**	**Girls**	**Boys**	**Girls**	**Boys**	**Girls**	**Boys**	**Girls**	
4	0	0	43	43	35	32	65	61	90	62	79	74	584
5	0	0	54	47	21	25	47	44	57	73	34	45	447
6	0	0	73	92	14	12	42	39	41	49	44	41	447
7	19	8	104	86	57	56	8	10	2	1	76	57	484
8	7	14	54	56	32	26	4	6	26	27	31	25	308
9	35	44	47	55	59	67	9	17	25	33	11	8	410
10	43	70	49	47	65	71	63	99	30	23	23	52	635
11	25	35	26	37	109	84	64	87	22	41	26	25	581
12	14	27	6	6	14	23	22	26	13	15	82	59	307
Total	143	198	456	469	406	396	324	389	306	324	406	386	4203

### Sedentary behavior measurement

With reference to the relevant items of the “Last 7-d Sedentary Time Questionnaire” (18) and “Adolescent Sedentary Activity Questionnaire” (ASAQ) (19), combined with the “Seven-day Activity Record Form for Children and Adolescents” in this study, the “Chinese Children and Adolescents Sedentary Behavior Questionnaire” was initially formulated. One hundred forty-two participants were invited to complete the repeated survey with an interval of seven days, and on the basis of combining the results of the pre survey and expert opinions, the questionnaire items were deleted and improved. After that, 181 participants were surveyed repeatedly at intervals of seven to 14 days. The reliability and validity of the revised questionnaire was good. The Spearman correlation coefficient of the two tests was between 0.51 and 0.85, and both were significant. The intra group correlation coefficient ICC (Intra class correlation coefficient) was 0.43 to 0.75, which met the expectations of the study. The validity of the questionnaire was tested using the criterion validity. The ASAQ questionnaire was used as the criterion. Spearman's correlation test showed that the correlation coefficient was 0.786, and the difference was statistically significant.

The revised questionnaire mainly investigates the basic information of the participants, the time they spent in regular cultural courses and different types of sedentary behaviors in a week (generally divided into study days and weekends). The questionnaire contains four dimensions and a total of 12 questions, including study sedentary behaviors (homework, reading, extracurricular tutoring classes, online courses), screen sedentary behaviors (watching TV, using mobile phones, computers), transportation sedentary behaviors (taking buses, taxis, etc.) and cultural leisure sedentary behaviors (painting, playing musical instruments, etc.). Since the sedentary behavior time of traffic accounts for a small proportion in daily life, it was not analyzed. This study used 8 h as the threshold, and divided the sedentary behavior time ≥8 h into the high sedentary behavior group. In addition, a survey on sedentary behavior interruption (i.e., how often children and adolescents walk when they are engaged in the above sedentary activities) has also been added. In previous studies, the sedentary behavior interruption interval was divided into 15 min (20), 20 min (21, 22), 30 min (23), 60 min (24), and 85 min (25). In this study, the median value of 30 min in previous studies was used as the sedentary behavior interruption interval.

### Parental lifestyle measurement

The “Parents' Living Habits Questionnaire” was used to investigate parental occupations, education status, per capita monthly household income, weekly time spent by parents on various types of sedentary behaviors, parents' intervention on children's sedentary time, and restrictions on the use of electronic products.

### Eye habits and vision measurement

Referring to the items of the relevant questionnaires, according to the eye habits of Chinese children and adolescents, the “Chinese Children and Adolescents' Eye Habit Questionnaire” was compiled. The contents of the questionnaire include the use of eyes in school (including the frequency of desks and chairs, the frequency of eye exercises, etc.), the use of eyes outside the school (including reading and writing posture, vision examination and correction, etc.), and the eyesight of students and their parents. Due to the epidemic, we were unable to go to the locality to measure the participants' visual acuity, it was obtained through the school visual acuity examination, using the “Standard Logarithmic Visual Acuity Scale” (GB11533-2011) in accordance with the requirements of GB/T26343-2010 “Technical Specification for Student Health Examination.” In addition, according to the filled visual acuity, the visual acuity level is graded: if the unaided visual acuity of both the left and right eyes is 5.0, the visual acuity is normal; If the vision of any unaided eye in both eyes is <5.0, it is considered as poor vision; If the vision of left and right eyes is inconsistent, the one with lower vision shall prevail. The World Health Organization (WHO) recommends that children and adolescents spend no more than 2 h of screen time per day, therefore, spending more than 2 h a day on screen time is defined as excessive use.

### Physical activity measurement

The Physical Activity Rating Scale compiled and revised by Chinese scholar Liang Deqing et al. and Japanese scholar Kimio Hashimoto was used to investigate the physical activity of individuals in the past week (26). Each dimension corresponds to five levels, of which the time dimension is scored from zero to four points, and the other two dimensions are scored from one to five points. The calculation method is: exercise volume = time ^*^ intensity ^*^ frequency, the minimum score is 0 points, and the maximum score is 100 points. The test-retest reliability of the scale was 0.82. The physical activity rating scale of this study is divided into two parts. The first part investigates the level of in-class physical activity of children and adolescents in the past week, and the second part investigates the level of extra-curricular physical activity of children and adolescents in the past week.

### Statistical analyses

The data collected from the questionnaire were sorted and screened by Excel software, and the data were statistically analyzed by SPSS 25.0 software and LMS chart maker software. Descriptive statistics was used to analyze demographic variables, sedentary time and sedentary type distribution characteristics, high sedentary behavior detection rate and poor vision detection rate of children and adolescents; Non normal distribution data are expressed in the form of median ± interquartile range. The preliminary analysis of the data found that the time standard deviation of sedentary behavior of the total sample was large, reflecting the high degree of dispersion of the data. In this case, the representation of the mean was low. Therefore, the median was used as a substitute for the mean. Independent samples *t*-tests were used to analyze differences in various sedentary behaviors among children and adolescents of different genders, to test for differences in the types of sedentary time between children and adolescents with normal and poor vision as well as to analyze differences in physical activity levels between children and adolescents with normal and poor vision. One-way ANOVA was used to examine the differences in the types of sedentary behaviors among children and adolescents in different grades and the differences in the duration of sedentary time among children and adolescents in different regions. A χ^2^ test was used to analyze the differences in the detection rate of poor vision among children and adolescents with different sexes, grades, and the habit of sedentary interruption. Through correlation analysis and stepwise regression analysis, on the premise of excluding the influence of parental occupation, education status and other factors, a model is gradually established to test the correlation between parents' sedentary time and children's and adolescents' sedentary time. Finally, logistic regression was used to analyze the relationship between the duration of sedentary, intermittent habits and the detection rate of poor vision. *P* < 0.05 indicates significant difference, *P* < 0.01 indicates very significant difference, and *P* < 0.001 indicates extremely significant difference.

## Results

### Sedentary behavior of Chinese children and adolescents

#### Times of sedentary behavior

The results of the survey showed the average total sedentary time of children and adolescents in grades four to 12 in China is about 8.1 h/day, 8.75 h/day on weekdays, and 7.2 h/day on weekends. The sedentary time on weekdays was significantly longer than that on weekends (*p* < 0.001). The total sedentary behavior time and weekend sedentary behavior time of students in grades 4–12 showed significant sex differences. The total sedentary behavior time (*p* < 0.05) and weekend sedentary behavior time (*p* < 0.01) of girls were significantly higher than those of boys. There was no significant sex difference in sedentary behavior time on weekdays (SB-WE) (Table 2).

**Table 2:** Sedentary time of Chinese children and adolescents (min/d).

**Grade**	**N**	**SB**			**SB-WE**			**SB-WD**		
		**M**	**F**	**Total**	**M**	**F**	**Total**	**M**	**F**	**Total**
4	584	422.5 ± 177.6	414.3 ± 183.1	419.1 ± 177.8	429.5 ± 141.5	443.0 ± 133.5	438.0 ± 137.3	390.0 ± 266.3	387.5 ± 249.4	390.0 ± 254.4
5	447	445.8 ± 182.5	468.5 ± 177.9	453.0 ± 183.0	450.0 ± 146.5	455.5 ± 153.3	453.0 ± 150.0	390.0 ± 293.5	465.0 ± 257.5	435.0 ± 285.0
6	447	454.9 ± 174.6	447.5 ± 170.1	450.0 ± 175.7	455.9 ± 142.3	446.0 ± 138.3	452.0 ± 136.0	430.0 ± 245.0	420.0 ± 245.0	425.0 ± 250.0
7	484	498.5 ± 217.1	492.0 ± 211.3	495.4 ± 211.9	552.5 ± 155.5	550.8 ± 142.3	551.8 ± 147.8	437.5 ± 314.4	446.3 ± 338.1	442.5 ± 326.3
8	308	498.5 ± 217.4	525.4 ± 199.2	505.5 ± 206.7	523.7 ± 196.8	534.1 ± 189.0	531.5 ± 189.5	460.0 ± 332.5	492.5 ± 301.0	470.0 ± 315.0
9	410	500.0 ± 218.9	541.3 ± 207.1	526.8 ± 215.0	539.0 ± 161.8	569.3 ± 168.0	550.5 ± 177.8	480.0 ± 271.6	537.5 ± 310.0	493.8 ± 292.5
10	635	505.5 ± 200.5	498.5 ± 214.6	502.5 ± 207.0	571.0 ± 177.5	550.2 ± 155.5	555.6 ± 164.0	425.0 ± 304.8	420.0 ± 320.0	420.0 ± 310.0
11	581	521.8 ± 224.8	525.0 ± 223.3	524.3 ± 220.3	603.0 ± 163.6	585.0 ± 169.6	593.0 ± 166.0	450.0 ± 330.0	440.0 ± 326.0	440.0 ± 334.5
12	307	469.5 ± 186.5	506.7 ± 187.4	490.3 ± 195.0	558.0 ± 202.0	615.0 ± 179.3	586.6 ± 190.0	350.0 ± 300.0	402.3 ± 270.0	375.0 ± 270.0
Total	4,203	477.5 ± 207.4	489.8 ± 200.3	484.0 ± 204.1	521.0 ± 184.0	529.0 ± 186.4	525.0 ± 184.8	420.0 ± 285.0	435.0 ± 297.6	430.0 ± 300.0

(1) Interquartile ± interquartile range; (2) SB = total sedentary behavior time; (3) SB-WE = sedentary behavior time on weekdays; (4) SB-WD = sedentary behavior time on weekends (the same below).

**Figure 1:**
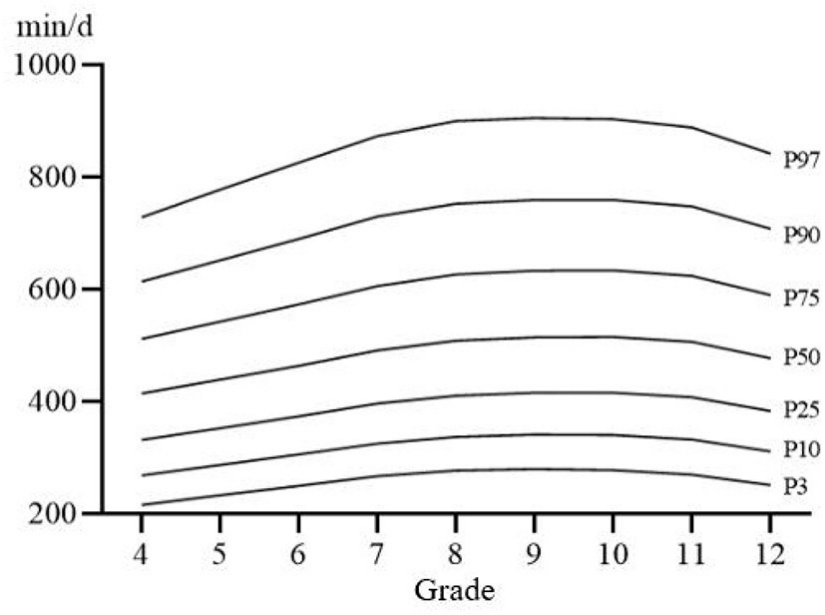
The percentile distribution curve of total sedentary time.

**Figure 2:**
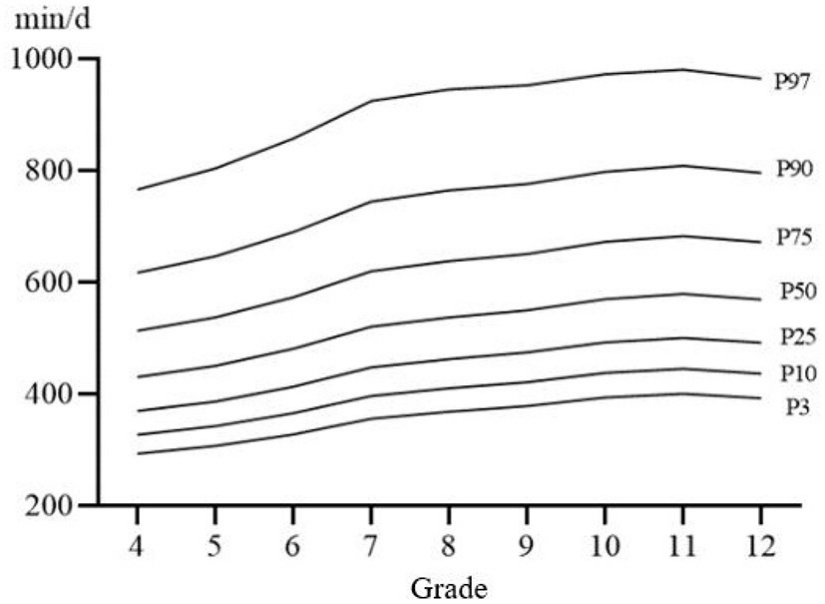
The percentile distribution curve of sedentary time in weekdays.

**Figure 3:**
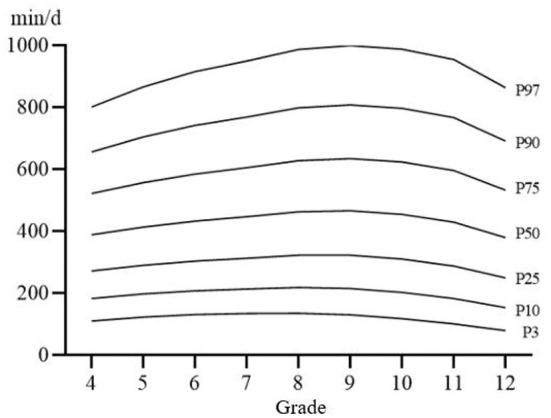
Distribution curve of percentile of sedentary time on weekends.

Figures 1–3 shows the percentile distribution of sedentary time of Chinese students from Percentile 3 (P3) to Percentile 97 (P97) and Table 3 shows the specific values of percentile sedentary time of Chinese students from P3 to P97, which generally show that children and adolescents in the upper grades have higher sedentary time. The sedentary time on weekends is longer in the upper grades than in the lower grades. The sedentary time on weekdays generally shows a trend of gradually increasing with the increase of grade, and reaches the highest point in Grade 11.

**Table 3:** Percentile distribution of sedentary time among Chinese children and adolescents.

	**Grade**	**L**	**M**	**S**	**P3**	**P10**	**P25**	**P50**	**P75**	**P90**	**P97**
SB	4	0.24	415.16	0.32	216.11	269.16	332.39	415.16	512.65	614.36	729.04
	5	0.17	439.45	0.32	233.6	287.8	353.01	439.45	542.87	652.57	778.38
	6	0.12	464.47	0.32	250.58	306.51	374.14	464.47	573.57	690.53	826.17
	7	0.1	491.68	0.31	267.37	325.93	396.82	491.68	606.55	730.1	873.89
	8	0.1	508.8	0.31	277.4	337.96	411.13	508.8	626.77	753.29	900.15
	9	0.13	514.98	0.31	280.14	341.9	416.24	514.98	633.5	759.8	905.43
	10	0.16	515.89	0.31	278.36	341.15	416.43	515.89	634.5	759.99	903.59
	11	0.19	506.92	0.32	270.32	333.02	408.06	506.92	624.36	748.04	888.89
	12	0.2	477.9	0.32	251.69	311.62	383.38	477.9	590.09	708.1	842.26
SB-WE	4	−0.86	430.90	0.24	293.76	327.56	370.01	430.90	513.52	617.47	766.47
	5	−0.87	451.11	0.24	307.39	342.78	387.25	451.11	537.89	647.29	804.57
	6	−0.87	481.39	0.24	328.34	366.03	413.40	481.39	573.77	690.22	857.62
	7	−0.88	521.13	0.24	356.62	397.18	448.09	521.13	620.30	745.23	924.75
	8	−0.88	537.74	0.24	369.33	410.96	463.13	537.74	638.66	765.17	945.75
	9	−0.86	550.01	0.23	379.27	421.70	474.67	550.01	651.12	776.53	952.99
	10	−0.83	570.04	0.23	394.27	438.21	492.83	570.04	672.72	798.57	972.70
	11	−0.80	579.62	0.23	400.80	445.68	501.30	579.62	683.16	809.03	981.27
	12	−0.78	569.80	0.23	392.89	437.31	492.35	569.80	672.03	796.02	965.07
SB-WD	4	0.57	388.55	0.48	110.10	182.97	272.23	388.55	522.02	656.19	801.22
	5	0.52	413.54	0.48	123.04	197.66	290.44	413.54	557.45	704.59	866.07
	6	0.49	432.71	0.48	131.42	208.00	304.02	432.71	584.78	741.80	915.64
	7	0.48	446.84	0.48	134.57	213.72	313.21	446.84	605.04	768.63	949.99
	8	0.50	462.96	0.49	135.79	218.76	323.08	462.96	628.14	798.49	986.82
	9	0.51	465.99	0.50	130.45	215.54	322.63	465.99	634.75	808.17	999.21
	10	0.53	454.27	0.51	118.04	202.86	310.27	454.27	623.64	797.35	988.28
	11	0.54	429.48	0.53	101.17	183.16	288.10	429.48	596.04	766.87	954.50
	12	0.55	379.54	0.55	79.95	153.78	249.59	379.54	533.12	690.84	864.10

In LMS analysis, L is lambda, that is power; M is mu, that is median; S is Sigma, that is coefficient of variation; P is Percentile, that is percentile.

According to one-way ANOVA, there was a significant difference in the total sedentary time of children and adolescents in different regions, East China > Northwest China > Northeast China > Southwest China > North China > Central and South China; The sedentary time on weekdays and weekends was also significantly different in different regions; Multiple comparisons showed that the sedentary time of children and adolescents in other regions on weekdays and weekends was significantly longer than that in central and southern regions (*p* < 0.001); The longest sitting time of weekdays was the highest in Northwest China (*p* < 0.05), and there was no difference between Northwest China and East China (*p* > 0.05); The sedentary time on weekends was the highest in East China, but there was no significant difference with southwest China (Table 4).

**Table 4:** Comparison of sedentary time among children and adolescents in different regions (min/d).

**Region**	**N**	**SB**	**SB-WE**	**SB-WD**
Northeast China	341	489.5 ± 192.0	527.0 ± 141.1	440.0 ± 315.0
North China	925	470.0 ± 220.1	510.0 ± 196.0	417.0 ± 305.0
East China	802	501.1 ± 203.1	553.1 ± 177.3	450.0 ± 298.1
Northwest China	713	501.0 ± 213.9	557.0 ± 209.4	435.0 ± 294.0
Southwest China	630	487.9 ± 187.6	507.5 ± 162.2	450.0 ± 274.3
South Central China	792	462.7 ± 195.4	487.0 ± 166.6	408.8 ± 300.0
Total	4,203	484.0 ± 204.1	525.0 ± 184.8	430.0 ± 300.0
F		10.596	21.655	3.055
P		0.001	0.001	0.006

#### Types of sedentary behaviors

In this study, study sedentary behaviors time, screen sedentary behaviors time, cultural leisure sedentary behaviors time and transportation sedentary behaviors time accounted for 79, 10, 9, and 2% of the total sedentary time in children and adolescents, respectively. The results showed that the sedentary time of study (*p* < 0.001), screen (*p* < 0.001) and transportation (*p* < 0.001) was significantly different between sex. The sedentary time of girls in learning (*p* < 0.001) and transportation (*p* < 0.05) was significantly higher than that of boys, and the sedentary time of boys in video was significantly higher than that of girls (*p* < 0.05). There was no difference between boys and girls in the sedentary time of cultural leisure.

The results showed that there were significant differences in study [*F*_(8,4202)_ = 270.674, *p* < 0.001], screen [*F*_(8,4202)_ = 7.458, *p* < 0.001], culture and leisure [*F*_(8,4202)_ = 18.070, *p* < 0.001] and transportation sedentary time [*F*_(8,4202)_ = 4.711, *p* < 0.001] in grades. The study sedentary time increased with the increase of grade. The study sedentary time in grades 11 and 12 was significantly higher than that in other grades (*p* < 0.001); The screen sedentary time of grade four and grade 12 was significantly lower than that of other grades (*p* < 0.001); The sedentary time of cultural leisure decreased with the increase of grade, and the sedentary time of cultural leisure in grade 12 was the lowest (Table 5).

**Table 5:** Sedentary behavior types of Chinese children and adolescents.

	**N**	**Studying**			**Screen**			**Leisure**			**Transportation**		
		**Boys**	**Girls**	**Total**	**Boys**	**Girls**	**Total**	**Boys**	**Girls**	**Total**	**Boys**	**Girls**	**Total**
4	584	380.0 ± 107.9	375.7 ± 108.2	380.0 ± 110.7	26.4 ± 49.1	25.7 ± 49.9	25.7 ± 49.8	46.1 ± 48.9	55.7 ± 52.5	51.4 ± 51.7	4.3 ± 15.7	7.1 ± 17.1	7.1 ± 17.1
5	447	380.0 ± 97.9	397.1 ± 114.1	389.3 ± 104.3	40.0 ± 72.9	42.8 ± 61.2	41.7 ± 64.3	51.4 ± 62.1	54.3 ± 48.6	54.3 ± 54.7	6.4 ± 18.6	4.3 ± 15.5	5.7 ± 17.1
6	447	393.6 ± 102.1	381.4 ± 97.9	387.9 ± 101.4	43.2 ± 72.0	42.9 ± 53.6	42.9 ± 58.5	45.7 ± 56.9	51.4 ± 57.1	49.6 ± 57.1	5.7 ± 18.6	8.6 ± 18.9	7.1 ± 18.6
7	484	478.7 ± 119.3	497.1 ± 131.6	489.1 ± 119.7	42.9 ± 80.4	33.9 ± 62.9	39.6 ± 70.6	38.6 ± 48.8	37.1 ± 44.3	38.6 ± 45.7	10.0 ± 21.4	10.7 ± 21.4	10.7 ± 21.4
8	308	483.9 ± 129.2	491.4 ± 137.1	486.1 ± 130.9	54.3 ± 83.4	40.0 ± 71.9	44.3 ± 76.8	33.9 ± 49.5	39.3 ± 48.6	36.1 ± 49.6	7.9 ± 20.4	8.6 ± 21.4	8.6 ± 21.4
9	410	491.4 ± 141.8	538.6 ± 138.9	522.1 ± 141.4	40.0 ± 71.8	38.6 ± 65.4	38.9 ± 68.6	24.6 ± 46.1	32.1 ± 46.1	30.0 ± 47.3	8.7 ± 20.4	10.7 ± 21.4	10.0 ± 21.4
10	635	504.0 ± 122.7	519.4 ± 115.4	512.1 ± 117.1	51.4 ± 78.6	35.7 ± 63.5	42.9 ± 70.7	30.7 ± 53.6	31.8 ± 43.2	31.4 ± 48.6	8.6 ± 21.4	9.4 ± 21.4	9.3 ± 21.4
11	581	521.4 ± 112.5	529.2 ± 122.9	525.0 ± 117.5	51.4 ± 92.7	40.0 ± 77.1	47.1 ± 82.1	35.0 ± 55.7	34.3 ± 47.9	34.3 ± 51.4	7.1 ± 17.1	10.7 ± 22.9	8.6 ± 20.0
12	307	499.3 ± 128.6	542.1 ± 143.9	528.6 ± 141.4	26.9 ± 60.0	30.7 ± 56.2	30.0 ± 56.3	21.5 ± 38.6	30.7 ± 36.8	28.6 ± 38.6	2.9 ± 17.1	10.0 ± 25.7	8.6 ± 21.3
Total	4,203	460.7 ± 141.2	477.3 ± 153.6	469.3 ± 148.6	40.7 ± 73.6	36.1 ± 59.2	38.6 ± 66.4	37.1 ± 53.8	40.0 ± 51.4	38.6 ± 53.1	7.1 ± 18.6	8.6 ± 21.4	8.6 ± 20.0

Children and adolescents in different regions also have significant differences in sedentary time in study, screen, cultural leisure and transportation. The study sedentary time in Northeast China was significantly higher than that in other regions (*p* < 0.001); The screen sedentary time in Northwest China was significantly higher than that in other regions (*p* < 0.001), and the screen sedentary time in Northeast China was the lowest (*p* < 0.05); The sedentary time of cultural leisure in Northwest and southwest regions was significantly higher than that in other regions (*p* < 0.001), and the sedentary time of cultural leisure in northeast region was significantly lower than that in other regions (*p* < 0.001); The sedentary time of traffic in Northeast China was significantly higher than that in other regions (*p* < 0.001) (Table 6).

**Table 6:** Comparison of sedentary types of children and adolescents in different regions.

	**N**	**Studying**	**Screen**	**Leisure**	**Transportation**
Northeast China	341	519.4 ± 112.1	17.1 ± 45.0	21.4 ± 28.2	17.1 ± 22.4
North China	925	442.9 ± 169.3	51.4 ± 78.6	42.9 ± 54.6	5.0 ± 17.1
East China	802	502.9 ± 169.3	34.3 ± 60.0	31.4 ± 41.6	8.6 ± 20.0
Northwest China	713	460.7 ± 143.9	64.3 ± 95.2	58.6 ± 63.6	7.4 ± 21.2
Southwest China	630	446.6 ± 140.9	33.6 ± 51.5	49.4 ± 60.0	10.7 ± 21.4
South Central China	792	460.0 ± 132.4	25.7 ± 48.6	34.3 ± 42.9	7.1 ± 17.1
F		51.708	75.646	47.129	22.688
P		0.001	0.001	0.001	0.001

#### Detection rate of high sedentary behavior

This study used 8 h as the threshold, and divided the sedentary behavior time ≥8 h into the high sedentary behavior group. Table 7 shows the distribution of the detection rate of high sedentary behavior in children and adolescents. The detection rate of high sedentary behavior in total sedentary time is 51.2%, the detection rate of high sedentary behavior on weekdays was 64.0%, and the detection rate of high sedentary behavior on weekends was 42.6%. The detection rate of high sedentary behavior on weekdays was significantly higher than that on weekends (*p* < 0.001).

**Table 7:** Detection rate of high sedentary behavior in children and adolescents of different sex [*N* (%)].

	**N**	**SB**		**SB-WE**		**SB-WD**	
		**<8 h**	**≥8 h**	**<8 h**	**≥8 h**	**<8 h**	**≥8 h**
Boys	2,041	1,037 (50.8)	1,004 (49.2)	780 (38.2)	1,261 (61.8)	1,187 (58.2)	854 (41.8)
Girls	2,162	1,015 (46.9)	1147 (53.1)	732 (33.9)	1,430 (66.1)	1,224 (56.6)	938 (43.4)
Total	4,203	2,052 (48.8)	2,151 (51.2)	1,512 (36.0)	2,691 (64.0)	2,411 (57.4)	1,792 (42.6)

There are significant differences in the detection rate of high and long sitting behavior among children and adolescents of different sex. The detection rate of high and long sitting behavior among girls was significantly higher than that of boys, both in the total sitting time and on weekdays (*p* < 0.01), and there was no significant sex difference in the detection rate of high sitting behavior on weekends (*p* > 0.05).

The detection rate of sedentary behavior in different grades was in the total sedentary time (*p* < 0.001), weekdays (*p* < 0.001) and sedentary time on weekends (*p* < 0.001), showing a trend of gradually increasing with the increase of grade. The detection rate of high sedentary behavior surged in Grade seven, but decreased significantly in grade 12 on weekends (Table 8).

**Table 8:** Detection rate of high sedentary behavior in children and adolescents of different grades [N (%)].

	**N**	**SB**		**SB-WE**		**SB-WD**	
		** <8 h**	**≥8 h**	** <8 h**	**≥8 h**	** <8 h**	**≥8 h**
4	584	392 (67.1)	192 (32.9)	385 (65.9)	199 (34.1)	385 (65.9)	199 (34.1)
5	447	258 (57.7)	189 (42.3)	263 (58.8)	184 (41.2)	256 (57.3)	191 (42.7)
6	447	271 (60.6)	176 (39.4)	263 (58.8)	184 (41.2)	274 (61.3)	173 (38.7)
7	484	219 (45.2)	265 (54.8)	118 (24.4)	366 (75.6)	266 (55.0)	218 (45.0)
8	308	123 (39.9)	185 (60.1)	104 (33.8)	204 (66.2)	158 (51.3)	150 (48.7)
9	410	151 (36.8)	259 (63.2)	110 (26.8)	300 (73.2)	189 (46.1)	221 (53.9)
10	635	278 (43.8)	357 (56.2)	117 (18.6)	518 (81.6)	357 (56.2)	278 (43.8)
11	581	219 (37.7)	362 (63.2)	87 (15.6)	494 (85.0)	318 (54.7)	263 (45.3)
12	307	141 (45.9)	141 (54.1)	65 (21.2)	242 (78.8)	208 (67.8)	99 (32.2)
Total	4,203	2,052 (48.8)	2,151 (51.2)	1,512 (36.0)	2,691 (64.0)	2,411 (57.4)	1,792 (42.6)

The detection rate of high sedentary behavior in different regions was shown in Table 9, and there are significant differences among different regions (*p* < 0.001). The detection rate of high sedentary behavior among students in East China was the highest (58.0%), followed by Northwest China (55.5%), and the lowest in South Central China (44.1%). There were also significant differences among various regions on school day (*p* < 0.001) and weekends (*p* < 0.01). The detection rate of high sitting behavior on school days was the highest in Northwest China (73.9%), and the lowest in South Central China (53.7%). The detection rate of high sedentary behavior on weekends was the highest in East China (47.0%), and the lowest in South Central China (38.1%).

**Table 9:** Detection rate of high sedentary behavior in children and adolescents in different regions [N (%)].

	**N**	**SB**		**SB-WE**		**SB-WD**	
		**<8 h**	**≥8 h**	**<8 h**	**≥8 h**	**<8 h**	**≥8 h**
Northeast China	341	163 (47.8)	178 (52.2)	90 (26.4)	251 (73.6)	187 (54.8)	154 (45.2)
North China	925	486 (52.5)	439 (47.5)	392 (42.4)	533 (57.6)	554 (59.9)	371 (40.1)
East China	802	317 (42.0)	4 65 (58.0)	226 (28.2)	576 (71.8)	425 (53.0)	377 (47.0)
Northwest China	713	317 (44.5)	396 (55.5)	186 (26.1)	527 (73.9)	415 (58.2)	298 (41.8)
Southwest China	630	306 (48.6)	324 (51.4)	251 (39.8)	379 (60.2)	340 (53.9)	290 (46.1)
South Central China	792	443 (55.9)	349 (44.1)	367 (46.3)	425 (53.7)	490 (61.9)	302 (38.1)
Total	4,203	2,052 (48.8)	2,151 (51.2)	1,512 (36.0)	2,691 (64.0)	2,411 (57.4)	1,792 (42.6)

**Figure 4:**
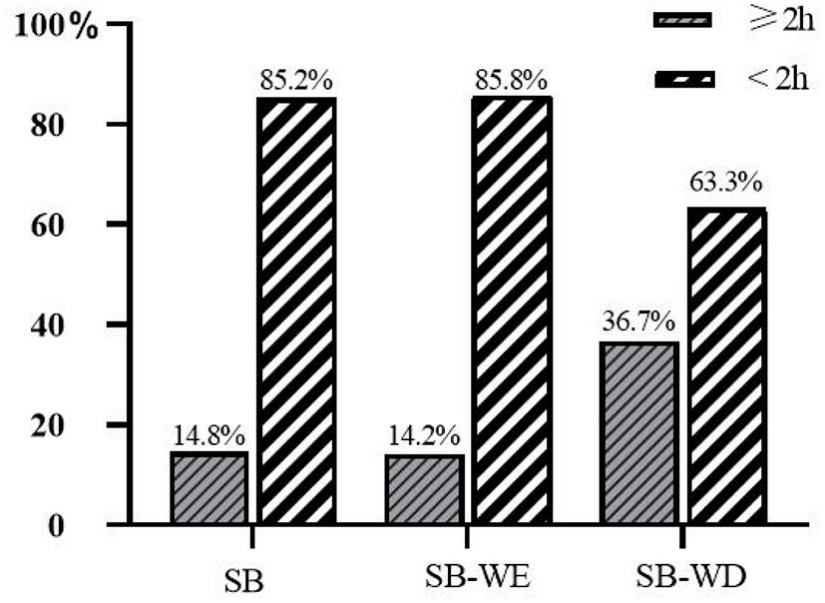
Detection rate of high sedentary behaviors in the screen category (%).

According to this standard, this study found that the compliance rates of Chinese children and adolescents in total screen-based sedentary time, screen-based sedentary time during weekdays, and screen-based sedentary time on weekends were 85.2, 85.8, and 63.3%, respectively (Figure 4).

#### Parental influence on children and adolescent's sedentary behaviors

The results in Table 10 show that the sedentary time of parents was significantly and positively correlated with the sedentary time of children and adolescents and various types of sedentary behavior. The intervention of sedentary behavior and the restriction of electronic products are significantly negatively correlated with the sedentary time of children and adolescents and the types of sedentary behavior.

**Table 10:** Correlation analysis of sedentary behavior in children and adolescents.

**Independent variable**	**Dependent variable**	**Correlation coefficient**	***p***
Total sedentary time	Parents' sedentary time	0.274	0.001
	Restrictions on the use of electronic products	−0.135	0.001
	Interventions for sedentary behavior	−0.083	0.001
Weekdays sedentary time	Parents' sedentary time	0.209	0.001
	Restrictions on the use of electronic products	−0.172	0.001
	Interventions for sedentary behavior	−0.100	0.001
Weekend sedentary time	Parents' sedentary time	0.230	0.001
	Restrictions on the use of electronic products	−0.069	0.001
	Interventions for sedentary behavior	−0.067	0.001
Study sedentary time	Parents' sedentary time	−0.157	0.001
	Restrictions on the use of electronic products	−0.110	0.001
	Interventions for sedentary behavior	0.055	0.001
Screen sedentary time	Parents' sedentary time	0.210	0.001
	Restrictions on the use of electronic products	−0.210	0.001
	Interventions for sedentary behavior	−0.026	0.046
Cultural leisure sedentary time	Parents' sedentary time	0.252	0.001
	Restrictions on the use of electronic products	−0.061	0.001
	Interventions for sedentary behavior	−0.054	0.001

Taking different types of sedentary time as dependent variables and the remaining three variables as independent variables, they were included in the multiple linear regression analysis. The regression results showed that the sedentary time of parents, the restrictions on the use of electronic products and the interventions for sedentary behavior significantly and differentially affected the sedentary time of children and adolescents (Table 11).

**Table 11:** Regression analysis of sedentary behavior in children and adolescents.

**Dependent variable**	**Independent variable**	**β**	**Standard error**	**Standardized β**	**t**	***p***	**ΔR^2^**
Total sedentary time	Parents' sedentary time	0.341	0.015	0.374	23.04	0.001	0.151
	Restrictions on the use of electronic products	−0.105	0.019	−0.088	−5.388	0.001	0.009
	Interventions for sedentary behavior	−0.226	0.069	−0.053	−3.272	0.001	0.003
weekdays sedentary time	Parents' sedentary time	0.249	0.015	0.278	16.434	0.001	0.087
	Restrictions on the use of electronic products	−0.139	0.02	−0.121	−7.126	0.001	0.017
	Interventions for sedentary behavior	−0.244	0.069	−0.059	−3.514	0.001	0.003
Weekend sedentary time	Parents' sedentary time	0.385	0.021	0.304	17.942	0.001	0.099
	Interventions for sedentary behavior	−0.476	0.053	−0.156	−8.99	0.001	0.031
	Restrictions on the use of electronic products	−0.07	0.027	−0.043	−2.543	0.011	0.002
Study sedentary time	Interventions for sedentary behavior	−0.476	0.053	−0.156	−8.99	0.001	0.031
	Parents' sedentary time	0.34	0.057	0.103	5.963	0.001	0.011
	Restrictions on the use of electronic products	−0.078	0.015	−0.091	−5.273	0.001	0.008
Screen sedentary time	Parents' sedentary time	0.133	0.009	0.26	15.391	0.001	0.072
	Restrictions on the use of electronic products	−0.06	0.007	−0.143	−8.399	0.001	0.092
	Interventions for sedentary behavior	−0.054	0.025	−0.036	−2.156	0.031	0.106
Cultural leisure sedentary time	Parents' sedentary time	0.331	0.016	0.352	21.282	0.001	0.124
	Interventions for sedentary behavior	−0.103	0.022	−0.076	−4.562	0.001	0.131
	Restrictions on the use of electronic products	−0.014	0.006	−0.038	−2.246	0.025	0.125

### Vision status and influencing factors of Chinese children and adolescents

#### Detection rate of poor vision in children and adolescents

It can be seen from Table 12 that the detection rate of poor vision of Chinese children and adolescents is 62.3%, and there are significant differences among different grades and sex. The detection rate of poor vision of girls is significantly higher than that of boys (*p* < 0.001). Higher detection rates of poor vision in upper grades, and the detection rate of poor vision is the highest in Grade 11 (*p* < 0.001).

**Table 12:** Detection rate of poor vision in children and adolescents.

**Grade**	**N**	**Boys**	**Girls**	**Detection rate**
4	584	140 (44.9)	120 (44.1)	260 (44.5)
5	447	100 (46.9)	128 (54.7)	228 (51.0)
6	447	110 (51.4)	139 (59.7)	249 (55.7)
7	484	151 (56.8)	138 (63.3)	289 (59.7)
8	308	96 (62.3)	98 (63.6)	194 (63.0)
9	410	121 (65.1)	160 (71.4)	281 (68.5)
10	635	188 (68.9)	268 (74.0)	456 (71.8)
11	581	196 (72.1)	241 (78.0)	437 (75.2)
12	307	107 (70.9)	119 (76.3)	226 (73.6)
Total	4,203	1,209 (59.2)	1,411 (65.3)	2,620 (62.3)

**Figure 5:**
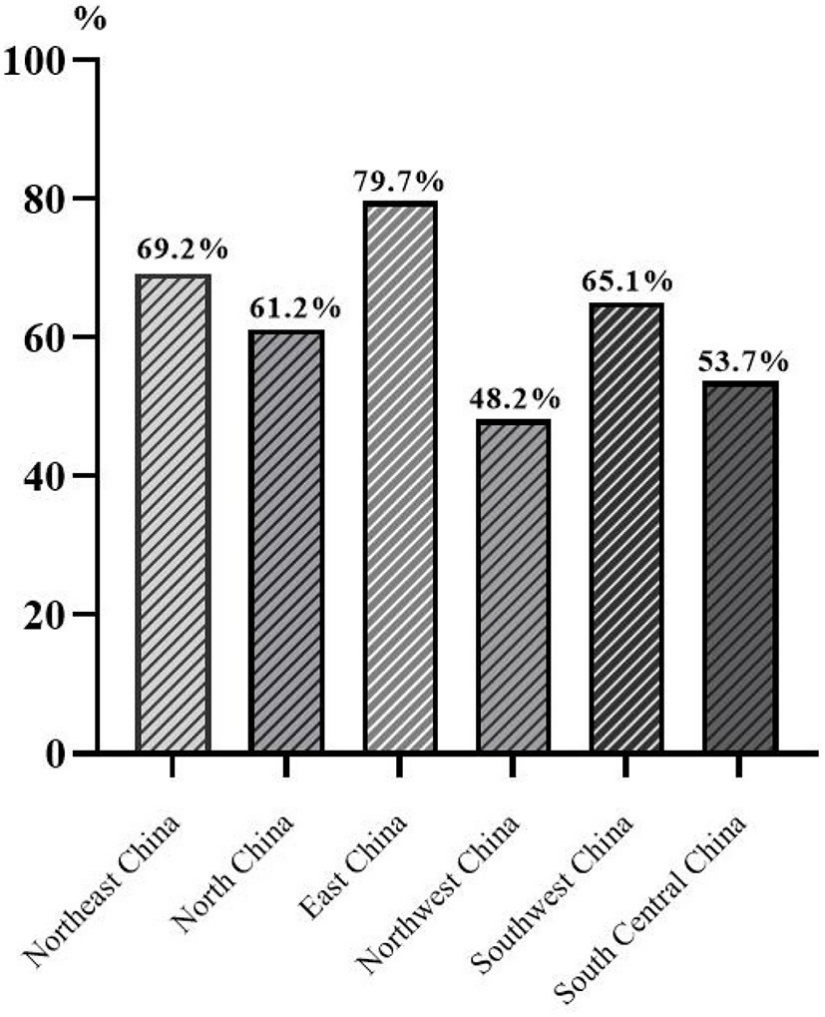
The detection rate of poor vision in children and adolescents by region.

In addition, there were also significant differences in the detection rate of poor vision between different regions (*p* < 0.001), with the highest in East China (79.7%) and the lowest in Northwest China (48.2%) (Figure 5).

#### Influencing factors of poor vision in children and adolescents

A questionnaire survey was conducted on the habit of using eyes, and it was found that habits including “Is your chest more than 6 cm from your desk?” (*p* < 0.001), “Are your eyes more than 33 cm from the books?” (*p* < 0.001), “Are your fingers 3 cm from the tip of the pen when you hold it?” (*p* < 0.001), “Whether the eyes are more than 66 cm away from the computer screen when using the computer?” (*p* < 0.001), “How many times a week do you do ocular gymnastics?” (*p* < 0.001), “Do you turn on a desk lamp or a roof lamp when you read or write at home after dark? Or do you use both?” (*p* < 0.001), and “During the past week, did you turn on the lights in the classroom? Or just turn on the lights when the weather was bad considering the lack of light?” (*p* < 0.001) are all important factors affecting the visual acuity of children and adolescents.

Genetics is also an important factor affecting poor vision. This study found that children and adolescents with both myopic parents had significantly higher rates of poor vision than children and adolescents whose parents had normal vision (*p* < 0.05).

The results show that the level of physical activity in class (*p* < 0.001) and the level of physical activity after class (*p* < 0.05) of children and adolescents with poor vision are significantly lower than those with good vision. Therefore, it can be seen that the level of physical activity will also affect the vision of children and adolescents.

### The relationship between sedentary behavior and poor vision in children and adolescents

#### The relationship between sedentary time and poor vision in children and adolescents

Table 13 presents the sedentary time of students with normal vision and those with poor vision. After normal transformation of the data and independent sample *t*-test, the results show that there is a significant difference between children and adolescents with normal vision and those with poor vision in sedentary time. Students with poor vision have significantly higher total sedentary time, school day sedentary time and weekend sedentary time than students with normal vision.

**Table 13:** Differences in sedentary time of children and adolescents with different vision conditions (min/d).

	**SB**	**SB-WE**	**SB-WD**
Normal vision	560.0 ± 198.6	506.0 ± 181.0	417.5 ± 300
Poor vision	591.4 ± 189.3	534.1 ± 185.5	440 ± 292.1

#### The relationship between sedentary types and poor vision in children and adolescents

The results showed that the sedentary time of students with poor vision was significantly higher than that of students with normal vision in study, screen, and cultural leisure, but there was no significant difference in sedentary time in transportation (Table 14).

**Table 14:** Comparison of sedentary time differences among students with different vision conditions.

	**Study**	**Screen**	**Transportation**	**Cultural leisure**
Normal vision	445.0 ± 145.0	36.4 ± 65.7	8.9 ± 21.4	35.7 ± 52.1
Poor vision	482.1 ± 146.4	42.9 ± 68.6	7.1 ± 17.8	41.4 ± 52.9

Taking writing posture, eye habits, parents' myopia, eye environment, physical activity level and other variables as covariates, the covariance method was used for analysis. indicating that excluding other influencing factors, study sedentary and screen sedentary still significantly affect the vision of children and adolescents, children and adolescents with poor vision have more sedentary time than normal vision in study and screen sedentary of children and adolescents. It can be seen in the binary logistic regression model that included poor vision as the dependent variable (Table 15), each unit of time spent in sedentary behavior in study and screen-based sedentary behaviors increased the risk of children and adolescents suffering from poor vision by 1.002 times.

**Table 15:** Binary logistic regression results of the effects of various sedentary time on visual acuity.

	**β**	**S.E**.	**Walds**	**df**	**Sig**.	**Exp (B)**
Study	0.002	0.001	20.485	1	0.000	1.002
Screen	0.002	0.001	14.218	1	0.000	1.002
Cultural leisure	0.001	0.001	1.437	1	0.231	1.001
Transportation	0.001	0.002	0.187	1	0.666	1.001

The relationship between sedentary behavior types and visual acuity was explored in different grades (Table 16). The results showed that in the primary school period, the screen sedentary time of students with poor vision was significantly higher than that of students with normal vision (*p* < 0.01); the sedentary time of students with poor vision in junior (*p* < 0.01) and senior high schools *p* < 0.001) was significantly higher than that of students with normal vision. Controlling for the influence of writing posture, eye habits, physical activity level and other variables, further covariance analysis found that screen sedentary still had a significant effect on primary school students' vision (*p* < 0.05), while in junior high school and senior high school, study sedentary was the main sedentary type affecting students' vision (*p* < 0.05).

**Table 16:** Comparison of sedentary types of students with different visual acuity conditions in different grades.

	**Primary school**		**Junior high school**		**Senior high school**	
	**Normal vision**	**Poor vision**	**Normal vision**	**Poor vision**	**Normal vision**	**Poor vision**
Study	380.0 ± 110.4	387.1 ± 102.5	485.0 ± 134.6	502.9 ± 131.25	503.8 ± 105.6	527.1 ± 127.9
Screen	30.0 ± 53.6	42.9 ± 60.0	40.0 ± 71.4	42.9 ± 40.0	39.3 ± 72.9	41.4 ± 79.4

#### The relationship between the detection rate of high sedentary behavior and the detection rate of poor vision in children and adolescents

In this study, the total sedentary time of children and adolescents was ≥8 h per day as high sedentary behavior. Table 17 shows the result of the difference in the detection rate of poor visual acuity. The analysis shows that the detection rate of poor vision of students with a total sedentary time of more than 8 h (65.7%) was significantly higher than that of students with a total sedentary time of <8 h (58.8%); The detection rate of poor vision of students who sat for more than 8 h on weekdays (65.5%) was significantly higher than that of students who sat for <8 h (56.7%); The detection rate of poor vision (64.8%) of students who sat more than 8 h on weekends was significantly higher than that of students who sat <8 h on weekends (60.5%).

**Table 17:** Differences in the detection rate of high sedentary behavior among children and adolescents with different vision conditions [*n* (%)].

	**Normal vision**	**Poor vision**	**Total**	***x^2^***
SB				
<8 h	845 (41.2)	1,207 (58.8)	2,052 (48.8)	21.109***
≥8 h	738 (34.3)	1,413 (65.7)	2,151 (51.2)	
SB-WE				
<8 h	655 (43.3)	857 (56.7)	1,512 (35.9)	32.184***
≥8 h	928 (34.5)	1,763 (65.5)	2,691 (64.1)	
SB-WD				
<8 h	952 (39.5)	1,459 (60.5)	2,411 (57.4)	7.997***
≥8 h	631 (35.2)	1,161 (64.8)	1,792 (42.6)	
Total	1,583 (37.7)	2,620 (62.3)	4,203 (100)	

#### Effects of sedentary behavior interruption on vision in children and adolescents

In this study, the median value of 30 min in previous studies was used as the sedentary behavior interruption interval. From Table 17, it can be seen that there are significant differences in the detection rate of poor vision in the combination of different sedentary time and interrupted habits (*p* < 0.001). Among them, the students whose sedentary time was more than 8 h without interrupting habits or interrupted once more than 30 min (BPS_1_) have poor vision detection (28.8%). Students whose sedentary time did not break once within 8 h and 30 min (BPS_4_) had the lowest detection rate of poor vision (21.1%), indicating that sedentary time and sedentary intermittent habits are important factors that significantly affect students' vision (Table 18).

**Table 18:** Differences in the detection rate of poor vision in children and adolescents with different sedentary habits.

	**BPS_1_**	**BPS_2_**	**BPS_3_**	**BPS_4_**	**Total**	***x^2^***	***p***
Normal vision	372 (23.5)	366 (23.1)	433 (27.4)	412 (26.0)	1,583 (37.7)	24.700	0.001
Poor vision	755 (28.8)	658 (25.1)	655 (25.0)	552 (21.1)	2,620 (62.3)		
Total	1,127 (26.8)	1,024 (24.4)	1,088 (25.9)	964 (22.9)	4,203 (100)		

Breaking up prolonged sitting (BPS); BPS1 = sedentary time more than 8 h without interruption or more than 30 min without interruption; BPS2 = sedentary time exceeds 8 h and breaks once within 30 min; BPS 3 = sedentary time not more than 8 h without interruption or more than 30 min without interruption; BPS 4 = sedentary time not more than 8 h, interrupted once within 30 min.

## Discussion

### Characteristics of sedentary behavior among Chinese children and adolescents

#### Significant differences in sedentary behavior among children and adolescents in different grades, sex and regions

From a global perspective, the sedentary time of children and adolescents in various countries has become a common problem. However, there are difference on sedentary time in children and adolescents among different countries. For instance, the average sedentary time of 13–17 years old adolescents in Spain is about 7.5 h per day (27), the average sedentary time of 12–17 years old adolescents in South Korea is about 9.5 h per day (27), and the average sedentary time of 9–10 years old children in European is about 9.6 h per day (3), the average sedentary time of 12-19 years old adolescents in the United States is about 6.9 h per day (2).

The results of this study show that the average total sedentary time of Chinese students in grades four to 12 is about 8.1±3.4 h/day. Studying sedentary behaviors such as class, homework, and reading are the main sedentary behaviors of Chinese children and adolescents, accounting for 79.2% of the total sedentary time. At present, the research on the sedentary behavior of Chinese children and adolescents is more consistent with the result that studying sedentary accounts for the largest proportion of total sedentary time. The participants of this study covered students from grades four to 12 in six administrative regions across the country. The survey results reflect the basic characteristics and changing laws of sedentary behaviors of Chinese children and adolescents to a certain extent.

Our study shows that Chinese children and adolescents in the upper grades have longer total sedentary behavior time and study sedentary behavior time, while screen sedentary behavior time and cultural leisure sedentary behavior time are shorter. This result is consistent with other studies (5). The reason for this result may be that as the grade increases, the academic workload increases, which leads to the students spending more time in and out of school for studying, doing homework and tutoring. This study found that although the change trend of sedentary time of male and female students was the same on the whole, the sedentary time of female students on weekends was significantly higher than that of male students. This may be due to the fact that students on weekends were far away from school constraints and had increased freedom in scheduling, which made the sedentary lifestyle of female students manifest. A study by Pereira et al. (28) also showed that boys spend slightly less sedentary time than girls. However, a systematic review of sedentary behavior tracking from childhood to adolescence abroad found that there was no significant sex difference in sedentary time between males and females (29). On the one hand, the retrospective process is pre-adolescence, and the difference between men and women has not been fully revealed, so the results are different from this study. The study also found that girls spend significantly more time studying on weekends than boys, and boys spend significantly more screen time than girls. The study by Guo et al. (6) also found that the sedentary time of screen sedentary in boys was slightly higher than that of girls, but they did not find a difference in total sedentary time.

#### Influence of family factors on sedentary behavior of children and adolescents

The family is an important place for children and adolescents to live and learn, and there is a close relationship between parents' living habits and children's sedentary behavior (30). This study found that the factors of influence were ranked according to standardized regression coefficients, with the order of importance being parents' sedentary time > restrictions on the use of electronic products > interventions for sedentary behavior. This shows that parental sedentary time has the greatest impact on the sedentary time of children and adolescents, and there is a significant positive correlation. Studies have pointed out that parents' intervention on children and adolescents' sedentary time also plays a very important role in shaping their healthy behaviors (31). Velazquez-Romero et al. (32) also believe that in today's increasingly popular models such as computers and smartphones, it is very important to set time limits on the use of electronic products. The results of this study are consistent with previous studies, that is, parents restricting children and adolescents' sedentary behavior and electronic product use time can effectively reduce children's and adolescents' sedentary time.

### The relationship between sedentary behavior and poor vision in Chinese children and adolescents

#### Sedentary time in children and adolescents is closely related to the detection rate of poor vision

In the World Health Organization's 2020 Guidelines on Physical Activity and Sedentary Behavior, it is pointed out that the longer the sedentary time is, the greater the harm to human health (33), and the same is undoubtedly true for the eyes and vision health of children and adolescents. This study found that both weekdays and weekends, children and adolescents with vision impairment were significantly more sedentary than children and adolescents with normal vision. A cohort study by Guggenheim et al. (34) also pointed out that the increased sedentary time has a hazard ratio of 1.17 for poor vision, that is, the longer the sedentary time, the higher the risk of developing poor vision. In addition, this study also found that the sedentary time and the detection rate of poor vision in Chinese children and adolescents have the same developmental characteristics, that is, both show that the higher the grade, the longer the sedentary time. This may be due to the increased schoolwork burden as the grade increases, resulting in increased sedentary time and longer eye-use time, resulting in decreased visual acuity in children and adolescents (35). Therefore, in order to protect the visual health of children and adolescents, it is necessary to reduce the academic burden of students, so as to fundamentally change the situation of sedentary time and improve students' eye habits.

In this survey, it was found that the detection rate of poor vision in children and adolescents with an average sedentary time of more than 8 h per day was significantly higher than that of children and adolescents with <8 h of sedentary time, and showed the same characteristics on weekdays and weekends. It can be seen that the daily sedentary time of more than 8 h will significantly affect the visual health of children and adolescents. At present, there are few studies on children and adolescents with high sedentary behavior, and there is a lack of specific countermeasures. Therefore, it is necessary to increase research on children and adolescents with high sedentary behavior in the later stage, and introduce corresponding preventive and intervention measures.

#### Study and screen sedentary are the main types of sedentary behaviors that affect poor vision in Chinese children and adolescents

This study shows that study and screen sedentary are the two main types of sedentary behaviors among children and adolescents in China, and for each additional unit of sedentary behavior in these two types of sedentary behavior, the risk of children and adolescents suffering from poor vision increases by 1.002 times. After excluding other factors that affect visual acuity, such as writing posture, eye habits, eye environment, parental myopia, and physical activity level, this study found that study and screen sedentary are still significant factors affecting children's and adolescents' vision.

Asian students have very heavy academic pressure, they are in a competitive learning environment from an early age and spend a lot of time on their studies, resulting in excessive eye use from an early age. A recent study in Japan found that the rate of poor eyesight among primary school students reached 75.6 and 95.9% among junior high school students (36). In China, a common occurrence in schools is that as students' homework time increases, children and adolescents' physical activity time and sleep time are significantly reduced, and the detection rate of poor vision increases significantly. However, poor vision may lead to poor academic performance (37). It is possible to yield twice the result with half the effort by adopting a scientific and reasonable study schedule and maintaining healthy and effective study and living habits.

Screen sedentary behavior is also an important factor affecting the eyesight health of children and adolescents. Studies have shown that the increased use of electronic devices by children and adolescents increases the risk of visual impairment (35). The results of a study in Taiwan also pointed out that children's addiction to screen entertainment has a negative impact on eye health (38). The WHO recommends that children and adolescents spend no more than 2 h of screen time per day (33). A study of the visual health of Danish adolescents also noted that adolescents who used screen devices for <2 h per day had better vision (39). According to this standard, 85.2% of children and adolescents in China in this study reached the standard. In Western countries, including the United States (40), Australia (41), Brazil (42) and other countries (43), only one third of children met the daily screen time recommendation of <2 h in the past decade. In the background of the widespread use of mobile electronic devices such as cell phones and computers has become the main way of learning, communicating or playing for children and adolescents (43). Health education programmes should emphasize the importance of limiting children's screen time, which will benefit children's eye health. Not only China, but also research reports from Europe (44), North America (45), and the Czech Republic (46). In view of the independent effects of screen sedentary on children and adolescents with poor vision, schools should reduce the use of electronic products in the teaching environment, and parents should limit the time children use electronic products.

This study further found that the poor vision of students in various stages was affected by different types of sedentary behavior. In primary school, the eyesight condition of students was largely affected by sedentary behavior of screen, and in junior high school and senior high school, study sedentary behavior had a greater impact on the eyesight level of students. Therefore, in order to protect the visual health of children and adolescents, parents, schools, society and the government should join hands to jointly monitor the sedentary time of children and adolescents in study and screen, and jointly protect the visual health of children and adolescents in China.

#### Interruption of sedentary behavior positively affects vision health

Continued sedentary time can adversely affect the body. Therefore, people should try to avoid prolonged sedentary behaviors in their lives and reduce the accumulation of sedentary time. This study found that children and adolescents whose sedentary time was interrupted once within 30 min and whose total sedentary time did not exceed 8 h a day had a significantly lower detection rate of poor vision than those who had accumulated sedentary time of more than 8 h without interruption or continuous sedentary Children and adolescents with an interruption after a time >30 min. Jenny (47) also pointed out that children who read continuously for more than 30 minu are more likely to have poor vision than children who read <30 min. Therefore, it is speculated that an interruption after 30 min of sedentary time is beneficial to reduce the rate of poor vision in children and adolescents. Studies have also shown that after a long period of sitting for a long time, intermittently every 30 min, standing for 2–3 min or short-term outdoor activities in the middle, can improve human metabolism (48), reduce self-fatigue (49), and reduce the risk of cardiovascular disease (20). In addition, staying indoors for too long is also detrimental to the visual development of children and adolescents (50). Therefore, going outdoors during sedentary breaks is a good way to rest, and this method has been proven to have a positive effect on children and adolescents' vision (51). Chastin (48) and Wennberg (49) both noted that breaking prolonged periods of sedentary activity every 30 min, coupled with 2–3 min of standing or low-intensity physical activity can alleviate feelings of fatigue and improve metabolic status. Considering with the current sedentary behavior of children and adolescents, 2–3 min of low-intensity walking as a sedentary break is more achievable and beneficial.

## Conclusion

(1) The average sedentary time of children and adolescents in grades four to 12 in China is about 8.1 h a day, of which study sedentary accounts for the highest proportion; With the increase of grade, the total sedentary time and study sedentary time gradually increased, and the sedentary time of screen and cultural leisure gradually decreased; There are significant sex differences in the total sedentary behaviors time and weekend sedentary behaviors time of Chinese children and adolescents, and girls are higher than boys; There are also significant differences in sedentary behaviors time among different regions, and the total sedentary behaviors time in East China is the highest.

(2) Reducing the sedentary time of parents and limiting the sedentary behavior of children and adolescents and the use of electronic products can effectively reduce the sedentary behavior of Chinese children and adolescents.

(3) The sedentary time of Chinese children and adolescents is closely related to the detection rate of poor vision; study and screen sedentary time are independent factors affecting the visual health of Chinese children and adolescents; The detection rate of poor vision in primary school is mainly affected by screen sedentary behavior, while the detection rate of poor vision in junior high school is mainly affected by study sedentary behavior.

(4) The interruption of sedentary behavior plays a positive role in visual health. Children and adolescents who have been sedentary for no more than 8 h a day and have interrupted behavior once after 30 min of sedentary behavior have a significantly lower risk of poor vision than children and adolescents who have been sedentary for more than 8 h a day and have been sedentary for more than 30 min.

## Strengths and key limitations

This study has a few limitations and strengths. There are several strengths over previous studies that need to be emphasized. On the one hand, the collection of participants in previous studies on sedentary behavior and poor vision in Chinese children and adolescents was limited to certein province and city, or even to a school. The study sample was not representative enough to reflect the current situation of sedentary behavior and visual health of Chinese children and adolescents. The participants in this study included children and adolescents in grades four to 12 in six major administrative regions of China. The study sample covers a wide range of regions and all administrative regions in China, and the age range includes children and adolescents who have the ability to fill in the questionnaire. On the other hand, compared with previous studies, this study not only investigated the relationship between the duration of sedentary behavior, type of sedentary behavior and poor vision of children and adolescents, but also explored the effect of interruption of sedentary behavior on poor vision of children and adolescents, and determined that sedentary behavior interruption is an indicator that cannot be ignored in the study of children and adolescents' visual health.

Several limitations of this study warrant noting. First, the visual acuity data collection was based on the report of the school visual acuity examination and completed by the participants through filing in the questionnaire without precise visual acuity assessment; Second, the study did not distinguish between urban and rural areas of residence of the participants, which may also be a variable affecting the relationship.

## Data availability statement

The raw data supporting the conclusions of this article will be made available by the authors, without undue reservation.

## Ethics statement

The studies involving human participants were reviewed and approved by East China Normal University. Written informed consent to participate in this study was provided by the participants' legal guardian/next of kin.

## Author contributions

LL and HF conceived the study and its design. JL and HF performed data analysis and drafted the initial manuscript. LL, JL, and BZ modified the manuscript. LL contributed to manuscript preparation, had full access to all aspects of the research, and writing process as well as primary responsibility for the final content. All authors contributed to the article and approved the submitted version.

## Funding

This study was supported by the Ministry of Education Humanities and Social Sciences Research General Program (19YJA890014). The funding bodies did not have a role in the design of the study, collection, analysis, data interpretation, and writing of this manuscript.

## Conflict of interest

The authors declare that the research was conducted in the absence of any commercial or financial relationships that could be construed as a potential conflict of interest.

## Publisher's note

All claims expressed in this article are solely those of the authors and do not necessarily represent those of their affiliated organizations, or those of the publisher, the editors and the reviewers. Any product that may be evaluated in this article, or claim that may be made by its manufacturer, is not guaranteed or endorsed by the publisher.
