# Supplementary material for: The association between sedentary behavioral characteristics and poor vision among Chinese children and adolescents
Source: Front Public Health. 2022 Dec 5;10:1043977. doi: 10.3389/fpubh.2022.1043977 (PMC9760758; doi:10.3389/fpubh.2022.1043977)
Supplement: Supplementary file 1 [file Data_Sheet_1.pdf]

儿童青少年生活习惯、体力活动水平及成年人生活习惯问卷

Children and adolescents living habits, physical activity level and adult living habits questionnaire

亲爱的家长和同学:
　　你们好！非常感谢你们在紧张的工作和学习之余填答问卷。本问卷主要调查当前青少年学生的生活习惯、身体活动和用眼情况，以及家长朋友们的生活习惯，答案无好坏之分，请尽可能真实准确地回答。你的认真填答是对我们最大的支持。数据仅用于学术研究，请放心填答。

Dear parents and students:
　　Hello! Thank you very much for completing this questionnaire in the midst of your busy work and study schedules. This questionnaire is designed to investigate the current habits, physical activity and eye use of young students, as well as the habits of their parents and friends. Your careful answers are our greatest support. The data will be used for academic research only, so please feel free to fill in your answers.

提示：本问卷分为两大部分，第一部分为学生问卷，由学生填答，第二部分为家长问卷，由家长填答。

Tips: This questionnaire is divided into two main parts, the first part is a student questionnaire to be filled by students, the second part is a parent questionnaire to be filled in by parents.

第一部分-学生填答

Part one-student fill in the answers

（一）基本信息

(1) basic information

1.你的姓名是: [填空题] *

1. Your name is: [fill in the blanks] *

____

2.性别: [单选题] *

2. Gender: [Multiple Choice Question] *

○男
O Male
○女
Women

3.请输入您的出生日期:　[填空题] *

Please enter your date of birth: [fill in the blanks] *

____

4.请选择城市: [填空题] *

4. Please choose your city: [fill in the blank] *

____

5.学校(请填写学校全称) [填空题] *

5. School (please fill in the full name of the school)[fill in the blank] *

____

6.年级(请保证填写正确的年级) * [单选题] *

6. Grade -LRB-please be sure to fill in the correct grade) * [single choice question] *

○一年级<br>Grade 1	○二年级<br>Grade 2	○三年级<br>Grade 3
○四年级<br>Grade 4	○五年级<br>Grade 5	○六年级<br>Grade 6
○七年级<br>Grade 7	○八年级<br>Grade 8	○九年级<br>Grade 9
○高一年级<br>Grade 10	○高二年级<br>Grade 11	○高三年级<br>Grade 12

7.你的班级是（举例：4 班） [填空题] *

7. Your class is (example: Class 4)[fill in the blank] *

____

8.你的视力水平为（视力五分法填写格式如：5.2）

8. Your visual acuity level is (the format for completing the visual acuity 5-point scale is as follow :5.2) *

*左眼 [填空题] *

Left Eye [fill in the blank] *

____

右眼 [填空题] *

Right Eye [fill in the blank] *

____

（二）看书及写作业时的用眼习惯

(2) the habit of using eyes when reading and doing homework

1.胸口是否离桌子超过 1 拳? [单选题] *

1. Is your chest more than 6 cm from your desk?? [single topic] *

○A.从不
Never
○B.偶尔
0b. occasionally
○C.经常
0c. frequently
○D.总是
0d. Always

2.眼睛是否距离书本超过 1 尺? [单选题] *

2. Are your eyes more than 33cm from the books? [single topic] *

○A.从不
Never
○B.偶尔
0b. occasionally
○C.经常
0c. frequently
○D.总是
0d. Always

3.手指是否距离笔尖 1 寸(约 3.3 厘米) [单选题] *

3. Are your fingers 3cm from the tip of the pen when you hold it? [single topic] *

○A.从不
Never
○B.偶尔
0b. occasionally
○C.经常
0c. frequently
○D.总是
0d. Always

4.你的父母或者老师是否提醒你的读写姿势不正确 [单选题] *

4. Do your parents or teachers warn you that you are not reading and writing correctly [single topic] *

○A.从不
Never
○B.偶尔
0b. occasionally
○C.经常
0c. frequently
○D.总是
0d. Always

5.你在天黑后看电子屏幕时开灯吗？ [单选题] *

5. Do you turn on the lights when you look at an electronic screen after dark? [single topic] *

○A.从不
Never
○B.偶尔
0b. occasionally

○C.经常
0c. frequently
○D.总是
0d. Always

6.你会躺着或趴着看书或电子屏幕吗？ [单选题] *

6. Do you read a book or an electronic screen on your back or stomach? [single topic] *

○A.从不
Never
○B.偶尔
0b. occasionally
○C.经常
0c. frequently
○D.总是
0d. Always

7.你在走路或乘车时看书或电子屏幕吗？ [单选题] *

7. Do you read a book or an electronic screen while walking or riding in a car? [single topic] *

○A.从不
Never
○B.偶尔
0b. occasionally
○C.经常
0c. frequently
○D.总是
0d. Always

8.你在用电脑时，眼睛距离电脑显示屏的距离超过 66 cm 吗？(约 3 个矿泉水瓶的长度) [单选题] *

8. Do you keep your eyes more than 66cm from the computer screen when using the computer? [single topic] *

○A.从不
Never
○B.偶尔
0b. occasionally
○C.经常
0c. frequently

○D.总是

0d. Always

9.在近距离用眼时，多长时间休息一次眼睛(远眺、闭目或户外活动等)？ [单选题]

9. How often do you take a break from using your eyes at close range (looking out, closing your eyes, outdoor activities, etc.) ? [single topic] *

○A. < 30 分钟

A.< 30 minutes

○B.30 分钟-1 小时

B. 30 minutes-1 hour

○C.1 小时-2 小时

C.1-2 hours

○D. > 2 小时

D .> 2 hours

10.在过去的一周里，一天做几次眼保健操？ [单选题] *

10. How many times a day did you do eye exercises in the past week? [single topic] *

○A.0 次

A.0 time

○B.1 次

B.1 time

○C.2 次

C.2 times

○D. > 3 次

D.> 3 times

11.你的父母是否近视？ [单选题] *

11. Are your parents near-sighted? [single topic] *

○A.父亲近视

A. The father is nearsighted

○B.母亲近视

B. The mother is nearsighted

○C.父母都近视

C. Both parents are nearsighted

○D.父母都不近视

D. Neither of the parents is near-sighted

12.你天黑后在家读书写字用什么灯光？ [单选题] *

12. Do you turn on a desk lamp or a roof lamp when you read or write at home after dark? Or do you use both? [single topic] *

○A.同时使用台灯和屋顶灯

A. Use both desk lamp and roof lamp

○B.仅使用台灯

B. Use desk lamps only

○C.仅使用屋顶灯

C. Use roof lights only

13.你过去一年内做过几次视力检查?

13. How many vision tests have you had in the past year? [single topic] *

○A.0 次

A.0 times

○B.1 次

B.1 time

○C.2 次

C.2 times

○D. > 3 次

D .> 3 times

14.过去一周里上课时教室开灯吗? [单选题] *

14. Have the lights been on in the classroom for the past week? [single topic] *

○A 不开(无论阴天或不使用投影时)

A. Not Open (whether cloudy or not using projection)

○B.不用投影时均打开

B. Open when no projection is needed

○C.只阴天上课时开

C. Only on cloudy days when classes are open

15.班级座位定时调换吗? [单选题] *

15. Do class seats change on a regular basis? [single topic] *

○A 从不(或仅个别人轮换)

A. Never (or only individual rotation)

○B.一学期一次

B. Once a semester

○一个月一次

C. Once a month

○D 每周一次

D. Once a week

16.学校按你的身高调整课桌椅高度吗？ [单选题] *

16. Does the school adjust the height of desks and chairs according to your height? [single topic] *

○A 从不或课桌椅不可调
A. Never or desks and chairs can not be adjusted
○B 一学年一次
B.Once every 2001 school year
○C 一学期一次
C. Once a semester
○D 两至三个月一次
D.Once every two to three months

17.你平均每天的睡眠时间为？格式：**小时**分钟 [填空题] *

17. What is your average daily sleep time? Format: * * hours * * minutes [fill in the blank] *

____

(三）生活习惯

(3) living habits

1. 过去 7 天，除上课时间外，你参与过下列活动吗？如果做过，写下你在这件事上花费的时间**（分钟/天）**，没有做过则填写“0”。（1 小时=60 分钟，2 小时=120 分钟，3 小时=180 分钟）

1. Have you participated in any of the following activities in the past 7 days except class time? If you have, write down the amount of time (minutes/day) you have spent doing it. If you haven't, write down"0". (1 hour = 60 minutes, 2 hours = 120 minutes, 3 hours = 180 minutes)

注意：空格里只能填写数字，单位为（分钟/天）

Note: Only numbers can be filled in the blanks, in units of (minutes/day)

周一-周五[表格数值题] [输入 0 到 600 的数字] *

Monday-friday [table number problem][enter numbers from 0 to 600] *

	星期一 Monday	星期二 Tuesday	星期三 Wednesday	星期四 Thursday	星期五 Friday
写作业 Do your homework					
阅读 Read					
课外文化课补习班 Extra-curricular remedial classes					
看电视 Watch TV					
乘坐公交车或私家车 Take a bus or private car					
练习乐器，听音乐,画画或手工					

频等）

Practice musical instruments, listen to music, draw or craft					
玩纸牌或下棋 Play cards or chess					
聊天 Chat					
使用电脑或手机学习 Use a computer or cell phone to study					
在电脑或手机上看视频（电影，电视剧，短视频等）					

Watch videos (movies, TV shows, short videos, etc.) on your computer or cell phone					
在电脑或手机上玩游戏 Play games on your computer or mobile phone					
在电脑或手机上聊天，刷微博等 Chat on the computer or mobile phone, scrolling					

micro-blog, etc.					

周末[表格数值题] [输入 0 到 600 的数字] *

Weekend [table problem][enter numbers from 0 to 600] *

	星期六 Saturday	星期天 Sunday
写作业 Do your homework		
阅读 Read		
课外文化课补习班 Extra-curricular remedial classes		
看电视 Watch TV		
乘坐公交车或私家车 Take a bus or		

private car		
练习乐器，听音乐,画画或手工 Practice musical instruments, listen to music, draw or craft		
玩纸牌或下棋 Play cards or chess		
聊天 Chat		
使用电脑或手机学习 Use a computer or cell phone to study		

在电脑或手机上看视频（电影，电视剧，短视频等）Watch videos (movies, TV shows, short videos, etc.) on your computer or cell phone		
在电脑或手机上玩游戏 Play games on your computer or mobile phone		

在电脑或手机上聊天，刷微博等 Chat on the computer or mobile phone, scrolling micro-blog, etc.		

2.你在进行上述活动时，平均间隔多久站起来走动一下？(例如，站起来，在某个地方散步，或者喝些饮料) [单选题] *

2. How often, on average, do you get up and move around when you do these activities? (Stand Up, take a walk somewhere, or have a drink, for example)

○A 0 分钟(不走动)
A.0 minutes (no walking)

○B 30 分钟左右
B.30 minutes or so

○C 45 分钟左右
C.45 minutes or so

○D1 小时左右
C.1 hours or so

（四）体力活动水平

(4) Level of physical activity

1.过去 7 天，你上过几节体育课？每节课多长时间？强度如何？

1. How many P.E. classes have you had in the last seven days? How Long is each class? How Strong is it?

	节数<br>Number of sessions	时间<br>Time	强度<br>Intensity
请填写<br>Please fill			

2.过去 7 天，除体育课外你参与体育锻炼的强度如何? [单选题] *

2. How much physical activity have you been doing outside of gym class for the past 7 days? [single topic] *

○A.轻微运动(比如散步、活动四肢这类)
A.Light exercise (such as walking or moving your limbs)

○B.强度不大的运动项目(更多的侧重娱乐性和放松性，例如做操、慢跑等)
B. Low-intensity sports (more focused on entertainment and relaxation, such as take execise, jogging, etc.)

○C.中等强度，需要较长时间的运动(比如骑行、长途跑步)
C. Moderate intensity, requiring long periods of exercise (such as cycling or long runs)

○D.呼吸加快、排汗量很大的运动项目，不过时间不会太久(例如篮球比赛踢足球等)
D. A sport in which breathing is faster and sweating is greater, but not for long (e.g. , basketball or soccer)

○E.呼吸加快、排汗量很大的持续性运动，参与时间比较长(例如长跑、游泳等)
E. Rapid breathing, perspiration of a large amount of continuous exercise, participation time is relatively long (such as long-distance running, swimming, etc.)

3.过去 7 天，除体育课外你参加以上的运动项目，一般一次多久? [单选题] *

3. For the past 7 days, how long have you participated in the above sports except physical education? [single topic] *

○A.<10 分钟
A.< 10 minutes

○B.11 - 20 分钟
B.11-20 minutes

○C.21 - 30 分钟
C. 21-30 minutes

○D.31 - 59 分钟
D.31-59 minutes

○E.>1 小时
E. > 1 hour

4.过去 7 天，除体育课外你参与以上体育锻炼的频率? [单选题] *

4. How often have you participated in the above exercises in the past 7 days, apart from Gym Class? [single topic] *

○A. 0 次
A.0 time

○B.1 次
B.1 time

○C.2 次
C.2 times

○D.3-5 次
D.3-5 times

○E.大于 7 次
E. More than 7 times

第二部分--家长填写

The second part- filled in by parents

1.您和孩子的亲属关系为 [单选题] *

1. What is your kinship with your child [single choice] *

○A.父亲
A. father

○B.母亲
B. mother

2.您的职业是（请在对应的数字上打勾√）: [单选题] *

2. Your occupation is (please tick the corresponding number) : [single-choice topic] *

○国家机关、党群组织、企业、事业单位负责人
Responsible Persons of state organs, party and mass organizations, enterprises and public institutions

○专业技术人员;如医生、护士、会计、教师、记者、科研人员等
Professional and technical personnel, such as doctors, nurses, accountants, teachers, journalists, scientific researchers and so on

○办事人员和文员;如机关、单位办事人员、文员、秘书、打字员、书记员
Clerks and clerks, such as office and unit clerks, clerks, secretaries, typists, clerks

○非技术性工人;如小贩、家务助理、邮递员、货运工人、清洁工人、包装工人，杂工
Non-skilled workers such as hawkers, domestic helpers, postmen, freight workers, cleaning workers, packaging workers, handymen

○技术性工人;如建造业工人，电工、机床操作员、油漆工人，印刷工人、司机
Skilled workers, such as construction workers, electricians, machine tool operators, paint workers, printing workers, drivers

○销售及服务业;如推销员、店员、小店店主、厨师及服务员、理发师、导游、空乘人员
Sales and services, such as salesmen, shop assistants, shopkeepers, cooks and waiters, hairdressers, tour guides and flight attendants

○军人
Soldiers

○农、林、牧、渔、水利业生产人员
Producers of agriculture, forestry, animal husbandry, fisheries and water conservancy

○照顾家庭/家庭主妇
Taking care of a family/housewife

○其他:（请注明） ________________ *
Others: (please indicate) *

3.您的教育水平为 [单选题] *

3. Your education level is [single choice] *

○A.高中及以下<br>High school and below	○B.大专<br>Higher vocational colleges	○C.本科<br>Undergraduates	○D.硕士<br>Master	○E.博士<br>Doctor

4.您配偶的教育水平为 [单选题] *

4. Your Spouse's education level is [single choice] *

○A.高中及以下 High school and below	○B.大专 Higher vocational colleges	○C.本科 Undergraduates	○D.硕士 Master	○E.博士 Doctor

5.您的家庭人均月收入的范围是（元）? [单选题] *

5. What is the range of your family's per capita monthly income (yuan) ? [single topic] *

○A.2000 以下 A below 2000	○B.2000-3999 B 2000-3999	○C.4000-5999 C. 4000-5999	○D.6000-7999 D. 6000-7999
○E.8000-9999 E. 8000-9999	○F.10000-19999 F. 10000-19999	○G.20000-29999 G. 20000-29999	○H.30000 及以上 H. 30000 and above

6.过去 7 天，您在除工作时间外，参与过下列活动吗？如果做过，写下你在这件事上花费的时间，没有做过则填写“0”。

6. Have you participated in any of the following activities outside of work hours in the past 7 days? If you have, write down how much time you have spent on it. If you haven't, write down“0”.

注意：空格里只能填写数字，单位为（分钟/天）

Note: only numbers are allowed in the blanks, in minutes/days

周一-周五[表格数值题] [输入 0 到 600 的数字] *

Monday-friday [table values][enter numbers from 0 to 600] *

	星期一 Monday	星期二 Tuesday	星期三 Wednesday	星期四 Thursday	星期五 Friday
阅读 Read					
看电视 Watch TV					
乘坐公交车或者私					

家车 Take a bus or private car					
练习乐器，听音乐,画画或手工 Practice musical instruments, listen to music, draw or craft					
玩纸牌或下棋 Play cards or chess					
聊天 Chat					
辅导孩子做作业 Help the children with their					

homework					
使用电脑或手机工作（在家里加班）<br>Use a computer or cell phone to work (work overtime at home)					
在电脑或手机上看视频（电影，电视剧，短视频等）<br>Watch videos (movies, TV shows, short videos, etc.) on					

your computer or cell phone					
在电脑或手机上玩游戏 Play games on your computer or mobile phone					
在电脑或手机上聊天，刷微博等 Chat on the computer or mobile phone, scrolling micro-blog, etc.					

周末[表格数值题] [输入 0 到 600 的数字] *

Weekend [table problem][enter numbers from 0 to 600] *

	星期六<br>Saturday	星期天<br>Sunday
阅读<br>Read		
看电视<br>Watch TV		
乘坐公交车或者私家车的时间<br>Time spent by bus or car		
练习乐器，听音乐,画画或手工<br>Practice musical instruments, listen to music, draw or craft		

玩纸牌或下棋 Play cards or chess		
聊天 Chat		
辅导孩子做作业 Help the children with their homework		
使用电脑或手机工作（在家里加班） Use a computer or cell phone to work (work overtime at home)		
在电脑或手机上看		

视频（电影，电视剧，短视频等） Watch videos (movies, TV shows, short videos, etc.) on your computer or cell phone		
在电脑或手机上玩游戏 Play games on your computer or mobile phone		
在电脑或手机上聊天，刷微		

博等<br>Chat on the computer or mobile phone, scrolling micro-blog, etc.		

7.过去 7 天，您平均每天的睡眠时间是多少?

7. What is your average amount of sleep per day for the past 7 days?

周一 ~ 周五：____________

Monday-friday: ____________

周六 ~ 周日：____________ [填空题] *[fill in the blanks] *

Saturday-sunday: ____________

8.您或您的配偶是否会带着孩子参与体育活动? [单选题] *

8. Do you or your spouse take your children to sports activities? [single topic] *

○A.从不<br>A.Never	○B.偶尔<br>B. Occasionally	○C.经常<br>C. frequently	○D.总是<br>D. Always

9.您的孩子长时间坐着的时候，您是否会提醒他站起来走动? [单选题] *

9. When your child sits for a long time, do you remind him to get up and move around? [single topic] *

○A.是<br>A.yes	○B.否<br>B. No

10.如果会提醒，孩子坐了多长时间后，您会提醒一次?（分钟/次） [填空题] *

１１０. If so, how long will the child sit after you will remind once? (minutes/Time)[fill in the blank] *

____

11.在您孩子使用电子产品的过程中，您是否会提醒他距离电子产品不要太近? [单选题] *

11.Do you remind your child not to get too close to electronics when they are using them? [single topic] *

○A.从不<br>A.Never	○B.偶尔<br>B. occasionally	○C.经常<br>C. frequently	○D.总是<br>D.Always

12.您是否会对您的孩子使用电子产品的时间进行限制? [单选题] *

12. Do you have any limits on the amount of time your child can spend using electronics? [single topic] *

○A.是<br>A.yes	○B.否<br>B. No

13.如果限制的话时间是多长时间? （分钟/天 ） [填空题]

１３. What's the time limit? (minutes/days)[fill in the blanks]

____
